# Insights Gained from *P. falciparum* Cultivation in Modified Media

**DOI:** 10.1155/2013/363505

**Published:** 2013-07-09

**Authors:** Sanjay A. Desai

**Affiliations:** The Laboratory of Malaria and Vector Research, National Institute of Allergy and Infectious Diseases, National Institutes of Health, Rockville, MD 20852, USA

## Abstract

*In vitro* cultivation of *Plasmodium falciparum*, the agent of severe human malaria, has enabled advances in basic research and accelerated the development of new therapies. Since the introduction of *in vitro* parasite culture nearly 40 years ago, most workers have used a medium consisting of RPMI 1640 medium supplemented with lipids and hypoxanthine. While these standardized conditions yield robust parasite growth and facilitate comparison of results from different studies, they may also lead to implicit assumptions that limit future advances. Here, I review recent studies that used modified culture conditions to challenge these assumptions and explore parasite physiology. The findings are relevant to understanding *in vivo* parasite phenotypes and the prioritization of antimalarial targets.

## 1. Introduction

The development of *in vitro *culture for the human pathogen, *P. falciparum*, has led to advances in basic, clinical, and applied malaria research [1]. The importance of *in vitro *culture to these advances is apparent when one considers the comparatively primitive understanding of the other major human parasite, *P. vivax*, for which *in vitro *cultivation is not universally available [2, 3].

Continuous *in vitro *cultivation of asexual stages of *P. falciparum* generally requires human erythrocytes, a lipid source, and a synthetic medium that provides a suitable ionic environment and soluble nutrients. There has been much discussion about possible effects of donor erythrocyte genotype on the success of *P. falciparum* cultivation, motivated partly by studies showing blood group O confers improved survival from malaria infections [4]. Nevertheless, anecdotal observations suggest that few, if any, cultivated parasite clones are adversely affected by changes in ABO group or other erythrocyte antigens. There are also frequent concerns about the precise lipid source with pooled human serum and commercial lipid-rich bovine serum formulations as the most common choices [5]. In our experience, cost is the primary determinant with bovine serum formulations being less expensive. Interestingly, we and other groups have observed that clones cultivated on one source of lipids exhibit transient growth retardation when switched to the other source, suggesting parasite adaptation to culture conditions. 

The final component, a synthetic medium consisting of required salts and nutrients in culture-grade water, is typically RPMI 1640 as used in the initial studies by Trager and Jensen [1]. This formulation, developed originally for the cultivation of leukocytes [6], contains major cations and anions at physiological levels based on values in human plasma; it also provides nutritive solutes that include glucose, amino acids, and key vitamins. Early studies, using removal of individual nutrients from RPMI 1640, identified a subset required for *in vitro *parasite cultivation [7]; they also found that supplementation with a purine source, typically hypoxanthine significantly improved propagation [8].

A whole generation of malaria researchers has grown up using this standard RPMI 1640-based recipe, with little or no variation. This has led to several implicit assumptions about parasite biology and *in vivo *behavior of clinical isolates. This review will summarize recent findings that challenge these assumptions and provide new insights into parasite biology. While it has been an important tool in the study of this pathogen, the standard RPMI 1640 formulation may be a poor predictor of *in vivo *parasite growth. Importantly, estimated parasite growth IC_50_ values for antimalarial compounds may not adequately reflect the compound's prospects for *in vivo *efficacy, at least for some targets. Another critical assumption has been that the ionic requirements of the parasite resemble those of mammalian cells, which tolerate only a narrow range of Na^+^, K^+^, Cl^−^, and H^+^ concentrations. The second section of this review summarizes a recent study showing that this assumption is also invalid. 

## 2. Nutrient Provision

An important consideration in synthetic media formulations is the provision of nutrients for propagation and expansion of cultured cells. An established list of nutritive solutes—sugars, amino acids, vitamins, and nucleobases—is required by cultured cells to varying extents. These nutrient building blocks provide an energy source for cell metabolism and enable synthesis of macromolecules such as proteins, nucleic acids, and lipids. Recognizing the generally conserved biochemistry of higher organisms, early research sought to develop standardized culture formulations that maximize *in vitro *growth of the greatest number of cell lines. This led to the development of media such as RPMI 1640 [6], which contains supraphysiological levels of the common nutrients.  Table 1 compares the nutrient concentrations in RPMI 1640 to measured values in healthy donors from developed countries and shows that most ingredients are present at significantly higher concentrations in the synthetic medium. Presumably, high concentrations were intentionally used as they may satisfy the growth requirements of diverse cell types, whose demands are likely to vary significantly. Another argument for higher nutrient concentrations is that they may help compensate for the absence of certain growth promoting factors. These factors are presumably made in the body but are absent from media because they are yet unknown; even if known, some factors may be labile in culture or too costly for routine addition. Finally, because most of the small nutritive solutes in Table 1 are inexpensive and not overtly toxic to cell cultures, it may have been reasonable to include them at higher than necessary levels in standard media.

How does provision of nutrients at supraphysiological concentrations affect malaria parasite cultivation and our understanding of parasite physiology? The answer depends, in part, on knowing which nutrients are essential for parasite cultivation, as was examined initially by [7] and also tallied in Table 1. Interestingly, subsequent studies found that some of these essential nutrients can be removed without detriment to parasite growth [9, our unpublished studies]. This might reflect either differences in experimental protocols or changes in parasite phenotypes over the intervening decades of *in vitro *propagation, as discussed further below.

To explore possible effects of high nutrients concentrations, we recently compared parasite growth rates in the standard synthetic formulation (RPMI 1640 medium supplemented with hypoxanthine and a lipid source) to that in pooled human serum from healthy American donors. We found that cultures of common laboratory parasite clones expanded at 60–90% faster rates in the synthetic medium than in pooled serum [10]. One explanation for preferential growth in RPMI 1640 is that these clones have been cultivated in this medium for many years and may therefore have adapted to faster growth in synthetic formulations. Another possibility is that use of media with supraphysiological nutrient concentrations may produce artificially high parasite expansion rates. This may seem desirable to experimentalists who demand large amounts of parasite material for their research, but it also confuses our understanding of *in vivo *parasite behavior.

Because a primary motivation of basic malaria research is to identify parasite targets for therapeutic intervention, another important question is whether *in vitro *studies using RPMI 1640 can faithfully determine which parasite activities are essential for *in vivo *survival and growth. A key target that illustrates this problem is the plasmodial surface anion channel (PSAC). This ion channel localizes to the host erythrocyte membrane and is conserved in malaria parasites that infect rodents, birds, and primates [11, 12]. Similar channels are absent from other parasites and mammals [13, 14]. These findings, along with its broad permeability to various nutrients, have made PSAC an attractive antimalarial target for many years [15, 16]. However, inhibitors found through small and large screens produced relatively weak *in vitro *growth inhibition under standard conditions [17], raising doubts about whether drug discovery should be pursued against this target. We recently recognized that use of media containing supraphysiological nutrient concentrations may adversely affect these growth inhibition studies: channels blocked by reversible small molecule inhibitors may allow adequate nutrient uptake and parasite growth when external concentrations are artificially elevated. A somewhat more physiological medium was therefore designed and used to test this possibility. We surveyed the list of essential nutrients in standard media and found that many have established permeability via PSAC [18, 19]. We selected isoleucine, glutamine, and hypoxanthine for detailed studies and eventually devised a modified medium (termed PGIM for PSAC growth inhibition medium) with lower values of these three reagents in a medium otherwise identical to the standard RPMI 1640 formulation. When supplemented with dialyzed human serum, this new medium supported continuous growth of parasites. Interestingly, the growth rate was somewhat lower than that in the RPMI 1640-based medium but comparable to that in pooled human serum without addition of synthetic formulations (described above). Most importantly, growth inhibition testing of PSAC inhibitors revealed significantly improved efficacy against parasite growth in PGIM than in RPMI 1640, often by 100-fold or more [10]. This improved parasite killing was seen with PSAC inhibitors from diverse scaffolds and was not an artifact of the modified medium because antimalarial drugs that do not block PSAC had unchanged efficacy in PGIM. 

Standard *in vitro *cultivation conditions may also yield incorrect assessment of some other drug targets, especially those involved in nutrient utilization or regulation of parasite energy status [20, 21]. Another area where effects of the specific medium used may be important is in the interpretation of high-throughput screens for compounds that inhibit parasite growth. Several of these so-called “whole-cell” screens have been carried out to search for antimalarial drug leads [22–24], but all have used the same culture conditions and only a few established laboratory parasite lines. While these studies have identified starting points for drug discovery, the corresponding parasite targets are unknown. The additional uncertainty of whether potent activity under standard *in vitro* conditions reflects *in vivo* efficacy against clinical malaria makes pursuit of these compounds riskier.

A final issue with use of synthetic media having supraphysiological nutrient levels is that they may select for parasite clones that do not properly represent *ex vivo *phenotypes. It is well established that adaptation of clinical parasite isolates to *in vitro *culture is associated with initial decreases in parasitemia and significant genome-level deletion and/or rearrangement [25, 26]. These changes yield parasite subpopulations that grow rapidly and eventually become the favorite laboratory clones of researchers. It is not clear how use of synthetic media influences the diversity of parasite phenotypes accessible to basic researchers, but it is a safe bet that the available laboratory clones are an imperfect proxy for *in vivo *parasite genotypes and phenotypes. 

## 3. Ionic Composition of Media Used for *P. falciparum *Cultivation

Whereas supraphysiological levels of nutrients may have been too readily accepted, the concentrations of ions in media may have followed the physiological values a little too religiously. The concentrations of Na^+^, K^+^, Cl^−^, Ca^++^, Mg^++^, and H^+^ in RPMI 1640 all approximate their normal values in human plasma (Table 2). Other commonly used synthetic media recipes also strive to maintain near physiological concentrations of these ions. The reluctance to deviate from these concentrations probably resulted from the tightly defined demands of mammalian cell lines for extracellular ions. Deviations in K^+^ and Ca^++^ may be most lethal, but excitable cells are notorious for poor tolerance to changes in any of these major and minor ions. 

In malaria research, this adherence to physiological levels of inorganic ions has led to implicit assumptions about parasite ionic requirements. For example, workers have assumed that the parasite also prefers a high Na^+^, low K^+^ extracellular environment and that it could not be successfully cultivated in highly nonphysiological conditions. Apparently supporting this assumption, the parasite remodels its host erythrocyte ionic composition by raising Na^+^ and decreasing K^+^ from their normal intracellular values (~10 and 100 mM, resp.) to levels similar to those in host plasma [27, 28]. This host cell cation remodeling is a consequence of significant permeability of these cations via PSAC [29]; channel-mediated transport dissipates the normal gradients established by the Na^+^/K^+^ pump at the host membrane. This cation remodeling has been assumed to promote intracellular parasite development in several ways.

We recently examined these assumptions along with the parasite's requirement and utilization of key ions by developing nonphysiological sucrose-based media for parasite cultivation [30]. These studies found that Na^+^ can be reduced from 143 mM to 7 mM without adverse effect on parasite propagation. Further reductions produced stage-specific effects on intracellular parasite development and yielded an EC_50_ of 1.2 mM for Na^+^ (Table 2), a surprisingly low concentration when compared to the poor tolerance of mammalian cell lines to such manipulations. We also found that malaria parasites can be cultivated with a remarkably broad range of extracellular K^+^ and Cl^−^ concentrations. A medium termed 4suc:6KCl (containing 83 mM sucrose, 105 mM K^+^, 6.8 mM Na^+^, 66 mM Cl^−^, and nutrients) supported parasite growth at rates matching those in standard RPMI 1640 medium. Because naïve cultures expanded at normal rates immediately upon transfer to this medium, the parasite does not appear to require adaptation prior to expansion in this nonphysiological environment. We measured erythrocyte cation concentrations in trophozoite-stage infected cells after cultivation in 4suc:6KCl and found that host cation remodeling was fully prevented, consistent with passive channel-mediated movements of Na^+^ and K^+^ under physiological conditions. Because parasite growth was not measurably affected, these findings revealed that host cation remodeling is an unnecessary byproduct of PSAC activity. This channel appears instead to be critical for nutrient acquisition. 

Use of nonphysiological media for *in vitro *cultivation has provided a number of other insights into parasite biology. For example, reducing Na^+^ below the above EC_50_ value produced trophozoites with engorged digestive vacuoles, suggesting parasite regulation of this ion within its compartments and possible new targets for intervention. Extracellular K^+^ also appears to be needed, albeit at low levels; this finding is surprising because one would have thought the sizeable erythrocyte K^+^ stores could adequately fulfill parasite demand. At the other end of the spectrum, parasite tolerance of K^+^ concentrations up to 148 mM provides strong evidence against an essential role for K^+^ in merozoite maturation [31]. In another study using nonphysiological media, we found that parasites also have a broad tolerance to changes in extracellular pH [32], a desirable trait in light of the metabolic acidosis that often accompanies severe malaria [33]. Finally, through the use of sucrose as an osmoticant to replace salts in the media, we found that merozoite egress and invasion depend on a defined range of ionic strength values [30], suggesting electrostatic interactions between parasite macromolecules that may play critical roles in merozoite egress and invasion.

## 4. Conclusions

*In vitro* cultivation of *P. falciparum*, developed nearly 40 years ago, has enabled fundamental advances in both basic and clinical malaria research. Over that time, a single RPMI 1640-based medium has achieved near universal use, leading to a number of assumptions about *in vivo *parasite behavior, drug action, prioritization of parasite targets for future therapies, and parasite biology. Recent studies have used modifications to this standard medium to gain new insights into these and other questions. Additional manipulations of culture conditions are needed to identify new targets for therapeutic intervention and uncover conditions that permit cultivation of refractory plasmodial species.

## Figures and Tables

**Table 1:** Concentrations of nutrients in RPMI 1640 and human plasma. Human plasma values represent measured means from indicated studies. Where available, data is from fasting donors without vitamin supplementation. Nutrients listed as “essential” for *P. falciparum *cultivation are based on significant growth impairment upon isolated removal from RPMI 1640 [7, 34]. *Hypoxanthine is not present in RPMI 1640 but is typically added for parasite culture.

Nutrient	Concentration in RPMI 1640, mM	Concentration in human plasma, mM	Ref. for human plasma	Requirement for* P. falciparum *culture
L-Arginine	1.15	0.082	[35]	
L-Asparagine	0.378	0.048	[35]	
L-Aspartic acid	0.15	0.013	[36]	
L-Cysteine	0.416	0.114	[35]	Essential
L-Glutamic acid	0.136	0.053	[35]	Essential
L-Glutamine	2.05	0.611	[35]	Essential
Glycine	0.133	0.268	[35]	
L-Histidine	0.097	0.086	[35]	
Hydroxy-L-proline	0.153	0.018	[35]	
L-Isoleucine	0.381	0.074	[35]	Essential
L-Leucine	0.381	0.141	[35]	
L-Lysine	0.219	0.191	[35]	
L-Methionine	0.101	0.03	[35]	Essential
L-Phenylalanine	0.091	0.061	[35]	
L-Proline	0.174	0.204	[35]	Essential
L-Serine	0.285	0.121	[35]	
L-Threonine	0.168	0.15	[35]	
L-Tryptophan	0.024	0.55	[35]	
L-Tyrosine	0.111	0.067	[35]	Essential
L-Valine	0.171	0.23	[35]	
D-Biotin	0.00082	3.91*E* − 7	[37]	
Choline Chloride	0.0215	0.013	[38]	
Folic Acid	0.00227	2.5*E* − 7	[39]	
Myoinositol	0.194	0.024	[40]	
Niacinamide	0.0082	3.0*E* − 4	[41]	
p-Amino Benzoic Acid	0.0073	N/D		
D-Pantothenic Acid	0.000524	2.3*E* − 4	[42]	Essential
Pyridoxine	0.0049	1.14*E* − 4	[43]	
Riboflavin	0.00053	1.05*E* − 5	[44]	
Thiamine	0.003	1.16*E* − 5	[45]	
Vitamin B-12	3.7*E* − 06	3.1*E* − 7	[46]	
D-Glucose	11.1	5.5		Essential
Hypoxanthine*	0.03	4.0*E* − 4	[47]	Essential

**Table 2:** Concentrations of inorganic ions. Reference normal ranges for human plasma are tallied for comparison; plasma concentrations for Ca^++^ and Mg^++^ represent the free or ionized forms of these ions. The ranges tolerated by *P. falciparum *cultures are listed as EC_50_ values as reported [30, 32]. The pH values are unitless.

Ion	RPMI 1640 value, mM	Human plasma value, mM	Reference for human range	Range tolerated by *P. falciparum *cultures, mM
Na^+^	142.8	135–145		1.2 to >150
K^+^	5.4	3.7–5.1		0.17 to >148
Cl^−^	108.3	95–105		31 to ≥108
Ca^+2^	0.42	1.03–1.23	[48]	N/D
Mg^+2^	0.41	0.55 ± 0.05	[49]	N/D
NO_3_^−^	0.84	0.02	[50]	N/D
SO_4_^−2^	0.41	0.3	[51]	N/D
HPO_4_^−2^	5.64	0.8–1.5		0.6 to ≥5.64
HCO_3_^−^	28.6	18–23		N/D
pH	7.2	7.31–7.45		~6.5 to >8
